# Intron-based genomic editing: a highly efficient method for generating knockin zebrafish

**DOI:** 10.18632/oncotarget.4547

**Published:** 2015-06-19

**Authors:** Jia Li, Baibing Zhang, Jiwen Bu, Jiulin Du

**Affiliations:** ^1^ Institute of Neuroscience, State Key Laboratory of Neuroscience, CAS Center for Excellence in Brain Science, Shanghai Institutes for Biological Sciences, Chinese Academy of Sciences, Shanghai 200031, China; ^2^ School of Life Science and Technology, ShanghaiTech University, Shanghai 200031, China; ^3^ University of Chinese Academy of Sciences, Shanghai 200031, China

**Keywords:** knockin, CRISPR/Cas9, zebrafish

## Abstract

The TALEN and CRISPR/Cas9 nuclease systems have been extensively utilized in genomic engineering of model organisms. In zebrafish, the nuclease systems have been successfully applied in generating loss-of–function knockout lines. However, genome-specific knockin techniques in zebrafish are still at the beginning. In this perspective, we briefly summarize the recent progresses on knockin approaches in zebrafish with a special focus on the newly developed intron-based knockin method.

Knockin animals are versatile tools for biological research [1]. For example, to understand the role of lethal genes in post-embryonic functions, it is usually to use knockin animals carrying *LoxP* insertions at interested genomic loci to generate conditional knockout animals [2]. Knockin-mediated fluorescent protein tagging of specific cells or endogenous proteins offers a powerful approach to track the dynamics of these cells or proteins *in vivo* [3]. For neuroscience research, knockin strategy can be used to make various animal tools for monitoring or manipulating the activity of specific types of neurons via cell type-specific expression of the calcium ion indicators GCaMPs, or the optogenetic elements channelrhodopsin-2 and halorhodopin, respectively [4].

Zebrafish (*Danio rerio*) is an emerging vertebrate animal model for life science. Although loss-of-function genomic editing for zebrafish mediated by zinc finger nucleases (ZFNs), transcription activator-like effector nucleases (TALENs), or the type II bacterial clustered regularly interspaced short palindromic repeats (CRISPR)/CRISPR-associated (Cas) 9 system (CRISPR/Cas9) has been developed [5-9], knockin approach is still at the beginning. Lack of feasible knockin methods for inserting a large DNA sequence into specific genomic loci is becoming a bottleneck for zebrafish-relevant research.

Recently, we reported a newly established knockin method in *Cell Research* [10]. In this work, we took the advantage of the donor design used in homology-directed repair (HDR)-mediated knockin in mice and NHEJ (nonhomologous end joining)-mediated donor integration in cell cultures, and developed a CRISPR/Cas9-mediated efficient knockin strategy for zebrafish, which can be widely applied for labeling different cell types and tagging endogenous proteins. Using this method, we have specifically labelled dopaminergic neurons, serotoninergic neurons, glia cells and endothelial cells. And we have also succeeded in adding an EGFP tag to the C-terminal of endogenous glial fibrillary acidic protein [10].

Previously, although HDR-mediated knockin strategies were used to insert short DNA sequences and repair mutations in the zebrafish genome [11, 12], the insertion of a large DNA sequence (e.g., *EGFP* or *Gal4*) to a specific genome loci was still challenging. It was reported that the EGFP sequence was correctly integrated at the zebrafish *tyrosine hydroxylase* (*th*) locus through TALEN-mediated double-strand breaks (DSBs) and HDR, and the germline transmission efficiency was about 1.5% [13]. However, the targeted *th* gene was destroyed and the inserted *EGFP* failed to express [13] (Table 1).

**Table 1:** Comparison between reported knockin methods in zebrafish

Lab	integration mechanism	sgRNA location	donor type	insertion	mean rate of germline transmission	disadvantage	advantage	application
Du lab [10]	NHEJ	intron	plasmid	Gal4/ EGFP	~12%	plasmid backbone insertion	large fragment insertion/endogenous gene integrity maintenance/high feasibility	cell-type specific labeling/endogens protein labeling
Ekker lab [11]	HDR	exon	ssDNA	LoxP	~10%	short fragment insertion	LoxP knockin	generation of LoxP knockin
Nusslein-Volhard lab [12]	HDR	exon	plasmid	single base	~11%	short fragment insertion	Correction of mismatches	correction of mismatches
Zhang lab [13]	HDR	exon	Linearized DNA	EGFP	~1.5%	disruption of endogenous gene	large fragment insertion	cell-type specific labeling
Del Bene lab [16]	NHEJ	exon	plasmid	Gal4	~10%	disruption of endogenous gene	large fragment insertion/easy donor design	cell-type specific labeling
Higashijima lab [17]	NHEJ	promoter	plasmid	Gal4/ RFP	~12%	disruption of promoter/ plasmid backbone insertion	large fragment insertion/easy donor design	cell-type specific labeling
Kawahara lab [18]	NHEJ/HDR	exon	plasmid	EGFP	~15%	disruption of endogenous gene	large fragment insertion/no plasmid backbone insertion	cell-type specific labeling/endogens protein labeling

NHEJ is at least 10-fold more active than HR during early zebrafish development [14, 15]. This is critically important, because the period from one-cell to embryonic stages, within which knockin integration takes place, is only several hours for zebrafish. The high efficiency of NHEJ increases the rate of successful integration in such a short period. Therefore, NHEJ-based approaches should be in principle more suitable to be applied for donor integration. Meanwhile, unlike HR, NHEJ does not need the precise homology between the parent zebrafish and the targeting donor, avoiding time-consuming screening and genotyping of parent animals.

Two NHEJ-based knockin approaches were recently developed to insert the transcriptional transactivator *Gal4* and *EGFP* into zebrafish genomic loci with high efficiency [16, 17]. However, the integrity of targeted genes were disrupted as insertion events occurred within either the exon [16] or 5′ *Cis*-regulatory elements of targeted genes [17]. As targeted genes themselves have biological functions, these strategies inevitably contaminate subsequent studies. To minimize indel mutations that introduced at junction sites in exons by the NHEJ mechanism, a recent study made use of a specially designed donor plasmid, which contains two *GFP* sgRNA target sequences and two 40-bp homologous sequences flanking sgRNA target locus in the genome [18]. When the *GFP* sgRNA and the genome-specific sgRNA were coinjected, the plasmid backbone is removed and the rest part of the donor is integrated into the targeted locus. The two 40-bp homology arm sequences enable the precise repair in the junction sites with an efficiency up to 79%, probably through both NHEJ and HDR-involved mechanisms [18]. However, as the sgRNA target sequence must be excluded from the donor, the last 20-bp sequence in the targeted exon is missing in the integrated knockin genome, causing the deletion of the last several amino acids at the C-terminal and functional abnormality of the endogenous protein (Table 1).

In our knockin system, a sgRNA target is selected in an intron of targeted gene, and a DNA sequence spanning from the sgRNA target site to 3′ intergenic region of targeted genes is added in a donor plasmid as the homologous arm (Figure 1). As this strategy retains the full reading frame and both the 5′ and 3′ regulatory elements of targeted endogenous genes, the integrity of targeted genes are maintained after predicted forward ligation of the donor into the targeted locus, which is verified by the western blot and the functional assays [10]. In addition, as the intron design of sgRNA targets can avoid imprecise integration in exons caused by NHEJ, and all insertion events are in-frame, our intron method results in a three-fold increase in the knockin efficiency in comparison with exon-based methods. The adding of the right arm in donor plasmid - the 3′ intergenic region of targeted gene – is critically important. However, if a SV40 poly-A sequence rather than the 3′ intergenic sequence of the endogenous gene is used, no fluorescence can be detected in *th-P2A-EGFP* knockin and *gfap-P2A-EGFP* knockin (unpublished data). This suggests that the endogenous 3′ UTR in the knockin donor is critical for the expression of the targeted exogenous gene. For the donor construction, based on our experience, the length of arms does not matter, as long as the arm sequence meets the following requirements: 1) sgRNA must be included in the intron part of the left arm. 2) 3′ intergenic region containing the full 3′UTR sequence of the mRNA must be included in the right arm. The other key point that guarantees successful knockin is the cleavage efficiency of the sgRNA. Several sgRNAs can be designed to target the intron sequence in the left arm, and the sgRNA with the highest cutting efficiency should be chosen to perform knockin experiment.

**Figure 1:**
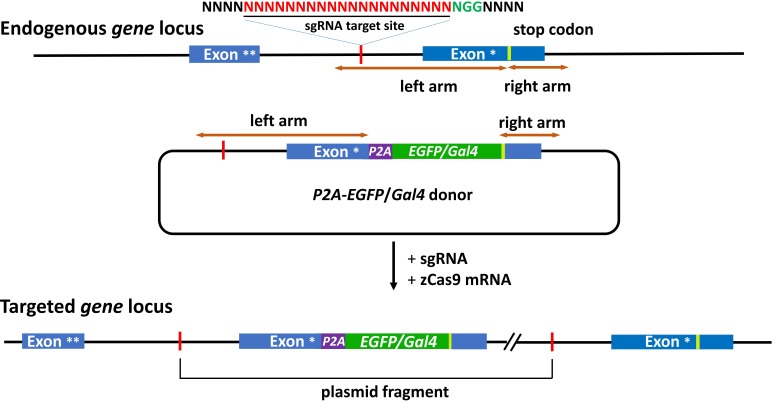
Schematic of the intron targeting-mediated strategy for generating *EGFP/Gal4* knockin at the zebrafish gene locus by using the CRISPR/Cas9 system The sgRNA target sequence is showed in red and the protospacer adjacent motif (PAM) sequence in green. The P2A peptide is a linker for multicistronic expression. The left and right arm sequences of the donor plasmids are indicated by the brown lines with double arrows. The *P2A-EGFP/Gal4* cassette was integrated into the endogenous *gene* locus after co-injection of the donor with the sgRNA and zCas9 mRNA. Exon ^*^, the last exon. Exon ^**^, the penultimate exon.

## OUTLOOK

In the near future, the next-generation knockin approach should be developed for tagging endogenous proteins. As the expression level of some endogenous proteins may be very low, knockin of one or two copies of fluorescent proteins is still insufficient for observation of these proteins *in vivo*. Moreover, adding more copies of fluorescent sequences in the targeted gene will decrease the transcriptional and expressional efficiencies. To solve these problems, we may integrate the intron-based knockin with other methods, such as SNAP-tag system, antibody-based fluorescent amplification system, and drug-induced fluorescent system. Taking advantage of the transparency of zebrafish larvae and super-resolution imaging, intron-based knockin approach will definitely open up a new avenue for zebrafish-relevant research.
